# Access to stroke rehabilitation services for patients discharged from the Mulago National Referral Hospital Neurology unit, Kampala—Uganda: a mixed-methods study

**DOI:** 10.3389/fstro.2026.1913292

**Published:** 2026-08-24

**Authors:** Ronald Bwambale, Ronald Ssenyonga, Mark Kaddumukasa, Martha Sajatovic, Aggrey David Mukose

**Affiliations:** 1 Department of Epidemiology and Biostatistics, School of Public Health, Makerere University, Kampala, Uganda; 2 Department of Public Health, Faculty of Health Science, Muni University, Arua, Uganda; 3 Department of Internal Medicine, College of Health Sciences, Makerere University, Kampala, Uganda; 4 Neurological and Behavioral Outcomes Center, University Hospitals Cleveland Medical Center and Case Western Reserve University School of Medicine, Cleveland, OH, United States

**Keywords:** access, experiences, rehabilitation, stroke, Thomas and Penchansky

## Abstract

**Introduction:**

Rehabilitation is the primary form of therapy for stroke survivors. However, there is a dearth of information regarding access to stroke rehabilitation services following discharge from hospitalization. This study, therefore, examined the level of access and experiences of accessing stroke rehabilitation services following discharge from the Mulago National Referral Hospital (MNRH) Neurology unit, Kampala, Uganda.

**Methodology:**

This was a concurrent mixed-methods study. Quantitative data were collected using a study questionnaire from 120 participants and analyzed with STATA version 15.0. A 17-item multidimensional tool was used to measure access, and participants who scored 80% and above were categorized as having high access. Qualitative data were collected through in-depth, face-to-face interviews with six purposively selected participants and analyzed thematically using ATLAS.ti version 25.

**Results:**

Of the 120 participants, 55.0% (66/120) were female, 55.8% (67/120) were married, and 44.2% (53/120) had attained a primary level of education. The overall mean age [Standard deviation (SD)] was 58.4 years (±14.6). Only 7.5% of the participants had high access to stroke rehabilitation services. Among the dimensions of access, acceptability scored highest (66.4%), followed by accessibility (24.3%), availability (20.6%), accommodation (15.9%), and affordability (3.7%). Key themes for experiences faced while accessing stroke rehabilitation services included transport challenges, high cost of care, adequacy of rehabilitation resources at hospitals, and organization of stroke rehabilitation services.

**Conclusion:**

Access to stroke rehabilitation services after hospital discharge remains suboptimal, with only a small proportion of patients achieving high levels of access. The high cost and limited affordability of rehabilitation care are the predominant barriers to service utilization.

## 1 Introduction

Stroke is among the leading causes of disability and the second leading cause of death worldwide, after ischemic heart disease (WHO, 2022). The World Health Organization (WHO) defines stroke as the rapid onset of focal or global neurological impairment of presumed vascular origin, with symptoms persisting for more than 24 h or resulting in death (Coupland et al., 2017). Globally, one person suffers a stroke every 3 s (WSO, 2022), causing over 86 million people to require rehabilitation (WHO, 2024a). Approximately 70% of global strokes and 87% of stroke-related disabilities occur in low- and middle-income countries (LMICs), highlighting the disproportionate burden of stroke in these settings (WHO, 2019). However, despite this high burden, these countries experience a 50% unmet need for rehabilitation (Gimigliano and Negrini, 2017). In Africa, stroke has an annual incidence rate of up to 316 per 100,000, and a 3-year fatality rate of over 80% (Akinyemi et al., 2021). Yet, at least 63% of people in need of rehabilitation care do not receive it (WHO, 2024b), despite global calls like Rehabilitation 2030, which advocate for available and accessible rehabilitation (Gimigliano and Negrini, 2017). The stroke burden is highest in sub-Saharan Africa (SSA), where it accounts for 11% of patient admissions (Limbole et al., 2022). In Ethiopia, over 38% to 79.3.3% of stroke survivors have limitations in basic and instrumental activities of daily living (Mohammed et al., 2023), and about half report long-term unmet supportive care needs (Tamrat et al., 2022). In Uganda, stroke is the sixth leading cause of death (Timm et al., 2023), and the third most diagnosed neurological condition, with a prevalence of 14.3% (Kaddumukasa et al., 2016), and a 30-day mortality rate of 38.1% (Olum et al., 2021), causing over 5,164 people to require rehabilitation each year (MOH, 2022). The Ministry of Health in Uganda reported limited research done about rehabilitation in the country (MOH, 2022), which leaves decision-makers with little or no data to inform policy or actions regarding rehabilitation. Previous studies carried out in Uganda highlighted low access to healthcare as a barrier to stroke recovery and prevention (Blixen et al., 2017), and a challenge of living with a stroke (Nakibuuka et al., 2021; Timm et al., 2023). However, these studies neither specifically evaluated access to stroke rehabilitation services nor quantitatively measured the level of access. Moreover, evidence on patients' experiences of accessing post-stroke rehabilitation remains limited. Therefore, this study assessed the level of access to and patients' experiences with stroke rehabilitation services following discharge from the Neurology Unit at MNRH.

## 2 Methods

### 2.1 Study design

This study used a concurrent mixed-methods design comprising a cross-sectional quantitative component and a qualitative descriptive component (Sandelowski, 2000). Quantitative and qualitative data were collected concurrently and independently, with integration occurring during participant selection, data reporting, and interpretation (Bell et al., 2022).

### 2.2 Study area

This study was conducted at Mulago National Referral Hospital-Neurology unit, along upper Mulago Hill Road, in Kawempe North Division of Kampala, the capital city of Uganda. MNRH is the largest tertiary care hospital in Uganda, with an official bed capacity of over 1,600. However, it has an actual capacity of 3,000 patients (Global Health Clinical Elective, 2019). The hospital serves as a teaching hospital for Makerere University College of Health Sciences and also provides specialized healthcare services in pediatrics, surgery, internal medicine services, diagnostics, research and training, and rehabilitation, with all their subspecialties and has both a private and a public wing (MNRH, 2024). The MNRH Neurology unit is one of the departments of MNRH that provides comprehensive care for neurological conditions and injuries. The unit records over 40–60 newly diagnosed stroke patients per month, who are initiated on rehabilitation care during admission. Since the unit receives patients from all over the country, discharged patients are recommended to receive outpatient rehabilitation care from nearby facilities that have the capacity to provide stroke rehabilitation care. The MNRH -Neurology unit is the central site for neurology care and research in Uganda; thus, it was the suitable site for examining access to stroke rehabilitation services for patients discharged from hospitalization.

### 2.3 Study population

The study population comprised adult stroke patients discharged from the MNRH-Neurology unit, with a recommendation to commence stroke rehabilitation care.

### 2.4 Inclusion criteria

The study included stroke patients aged ≥18 years, with at most 3 months post-discharge from the MNRH-neurology unit. This period was selected because the first 3 months after a stroke are the most important for recovery (Preeti, 2024).

### 2.5 Exclusion criteria

The study excluded patients without accessible mobile telephones and those that had died at the time of the study.

### 2.6 Sample size estimation

For the quantitative component, the study used Cochran's formula for a cross-sectional study where the outcome is a categorical variable. This formula uses a *Z*-score, an estimated proportion of the outcome, and the margin of error. Using a *Z*-score of 1.96, 5% margin of error and a prevalence of 26.4%, the proportion of people living with disabilities in Kawempe Division who utilized rehabilitation services (Zziwa et al., 2019).


$$\begin{array}{lll} n & = & \frac{Z^{2}*P*\left(1-P\right)}{\delta }\\ = & \frac{1.96*1.96*0.268*0.732}{0.05*0.05}\\ = & 302participants \end{array}$$

Accounting for a non-response rate of 5%.


$$\begin{array}{l} \text{Adjusted}n=\frac{n}{1-r}\\ \frac{302}{\left(1-0.05\right)}=312\;\text{participants} \end{array}$$

However, the MNRH neurology unit, on average, receives 50 newly diagnosed stroke patients per month; thus, the maximum number of patients attainable 3 months before the study and 1 month during data collection is 200 participants.

Adjusting for a finite population results into;


$$\begin{array}{ll} = & \frac{n}{1+\;\left(n/popn\right)}\\ = & \frac{312}{1+\;\left(312/200\right)}\\ = & 122participants \end{array}$$

The study proposed to enroll 122 participants.

For the qualitative component, participants were recruited until data saturation was achieved. Saturation was ascertained through daily debriefs between the principal investigator and research assistants. Six in-depth interviews were adequate to reach saturation (Guest et al., 2006).

### 2.8 Sampling procedure

For the quantitative component, the study used the consecutive sampling technique to recruit stroke patients who met the inclusion criteria and consented to participate. This approach was chosen due to the limited number of stroke patients registered at the unit, which could not permit an adequate sampling frame for a probabilistic sampling technique.

For the qualitative component, participants for in-depth interviews (IDIs) were purposively selected from respondents who had completed the quantitative survey. Priority was given to individuals who had attended at least half of the recommended rehabilitation sessions and consented to participate in interviews, as repeated exposure to rehabilitation services was expected to yield richer insights into access-related experiences.

### 2.9 Study variables

#### 2.9.1 The dependent variable

The dependent variable was access to stroke rehabilitation services for patients discharged from the MNRH Neurology unit, defined as the ability of a stroke patient to reach a health facility that could provide stroke rehabilitation services and find the services available, accessible, affordable, acceptable, and accommodating to their needs. This level was measured using a 17-item dimensional tool across the five dimensions of access: accessibility (4), availability (3), accommodation (4), affordability (4), and acceptability (2). Each item was rated on a Likert scale of five points (1–5), with one as the lowest, and five as the highest (Supplementary Table 1).

The total maximum score of a given dimension of access was obtained by adding the highest scores of all the items within that dimension. To determine how much (%) an individual performed in a particular dimension, their scores on each item within that specific dimension were added together, divided by the maximum total score of that dimension, and multiplied by 100. To determine an individual's overall composite score on access, all item scores from the five dimensions were summed together and turned into a percentage of the maximum total score of all dimensions.


$$\begin{array}{l} =\frac{\;sum\;of\;all\;item\;scores\;from\;the\;five\;dimensions}{maximum\;total\;score\;of\;all\;dimensions}\times \;100 \end{array}$$

In total, the minimum composite score was 20%, while the maximum composite score was 100%. A higher percentage implied higher access to stroke rehabilitation services. An overall composite score of 80% and above was categorized as high access, and a score below 80% was categorized as low access to stroke rehabilitation services. A cut-off percentage of 80% was chosen, following literature from previous scholars who examined access to health services among chronically ill older adults with physical disabilities in Thailand (Nanthamongkolchai et al., 2022). Statistically, this cut-off represented participants who achieved adequate access to stroke rehabilitation services across the five dimensions of access—availability, accessibility, acceptability, accommodation, and affordability.

#### 2.9.2 Independent variables

To measure the socio-economic status (SES), a wealth index score was determined from household assets as used by the Uganda Bureau of Statistics (UBOS) (UBOS, 2023). Assets assessed included ownership of a radio, television, mobile phone, bicycle, motorcycle, motor vehicle, a piece of land, a manufactured bed, plastered wall, cemented floor, and electricity. This information was summarized using principal component analysis. A principal component to which more assets loaded was used to represent SES. A high score on the principal component implied a higher level of SES. Using the mean of individual scores on the principal component, individuals were then classified into low and high SES. The type of residence was determined using administrative designations of an urban area: a city, municipality, town, or a trading center with more than 1,000 persons per square kilometer. Further information on the measurement of independent variables is provided in Supplementary Table 1.

### 2.10 Data collection

Phone contacts of stroke patients who were discharged from the inpatient department of the MNRH-Neurology unit and met the inclusion criteria were obtained from the medical discharge records of the unit. These were contacted for reachability and eligibility. A participant was considered inaccessible after five failed attempts to be reached through a mobile telephone (Soares et al., 2024). Eligible and reachable participants were informed about the study, and, if interested, were scheduled for an interview at a convenient time. For in-depth interviews, face-to-face interviews were arranged at the participants, homes. For aphasic participants, assisted interviews with a caretaker well-versed with the situation of the participant were conducted. For the quantitative component, telephone interviews were conducted using a structured questionnaire that consisted of two sections. The first section collected socio-demographic data; age, sex, marital status, religion, highest level of education, and area of residence, while the second section assessed the level of access using a 17-item multi-dimensional tool covering five dimensions of access: availability, affordability, accommodation, and acceptability. Items were rated on a Likert scale, with clear response instructions provided.

For the qualitative component, in-depth face-to-face interviews were conducted using a semi-structured IDI guide. The first section explored general understanding of stroke and rehabilitation services, while the second section focused on participants' experiences in accessing stroke rehabilitation services. Probes were included for each access dimension and the strategies used to cope with challenges faced while accessing stroke rehabilitation services. In-depth interviews lasted an average of 30 mins.

### 2.11 Quality control and assurance

Two research assistants were trained prior to data collection on the study objectives, methodology, interview techniques, and ethical considerations for both quantitative and qualitative components. The multi-dimensional tool underwent expert review and evaluation for internal consistency. A Cronbach's alpha of 0.83 was obtained, indicating that the tool was reliable in measuring access to stroke rehabilitation services (Mohd Arof et al., 2018). Informed consent forms were translated into Luganda, Kiswahili, Ateso, Runyakitara, and Luo. The questionnaire and the IDI guide were translated into Luganda, the commonly spoken local language. During data collection, questionnaires were checked for completeness before entering data into statistical packages.

### 2.12 Data management and analysis

Quantitative data from the structured questionnaires were entered into EpiData software for data cleaning and then exported to STATA version 15.0 for analysis. Data was stored on the personal computer of the principal investigator and backed up on Google Drive. Hard copies were kept in a locked cupboard for safety. Qualitative data from recordings of in-depth interviews and key informant interviews were concurrently translated to English and transcribed verbatim in Microsoft Word. All transcripts were checked for accuracy and completeness with verification from the recordings by the principal investigator before being exported to ATLAS.ti version 25 for analysis. Data was stored on the computer of the principal investigator with backup versions on Google Drive.

### 2.13 Data analysis

For quantitative data, descriptive statistics were presented as percentages and frequencies for categorical variables, while continuous variables were reported as means with standard deviations if data was normally distributed, and as medians with interquartile ranges if skewed. Normality of data distribution was statistically ascertained using the Shapiro–Wilk test. The proportion of participants that scored 80% and above on the multi-dimensional tool of access was determined. Missing data was managed using the complete-case analysis approach.

Qualitative data were analyzed using inductive thematic analysis (Braun and Clarke, 2006). Data from the recordings were transcribed verbatim to Microsoft Word and imported into ATLAS.ti. version 25 for analysis. The analysis followed the six-phase step guide by Braun and Clarke (2006). This included familiarization with data through reading the transcripts several times, coding, theme development, review, redefining and naming. Related subthemes were merged into themes. Representative quotations were selected for each subtheme. RB independently coded the transcripts and, together with ADM, reviewed, refined, and named the subthemes and themes. Neither RB nor ADM was involved in data collection.

## 3 Results

### 3.1 Socio-demographic characteristics of participants

**Figure 1:**
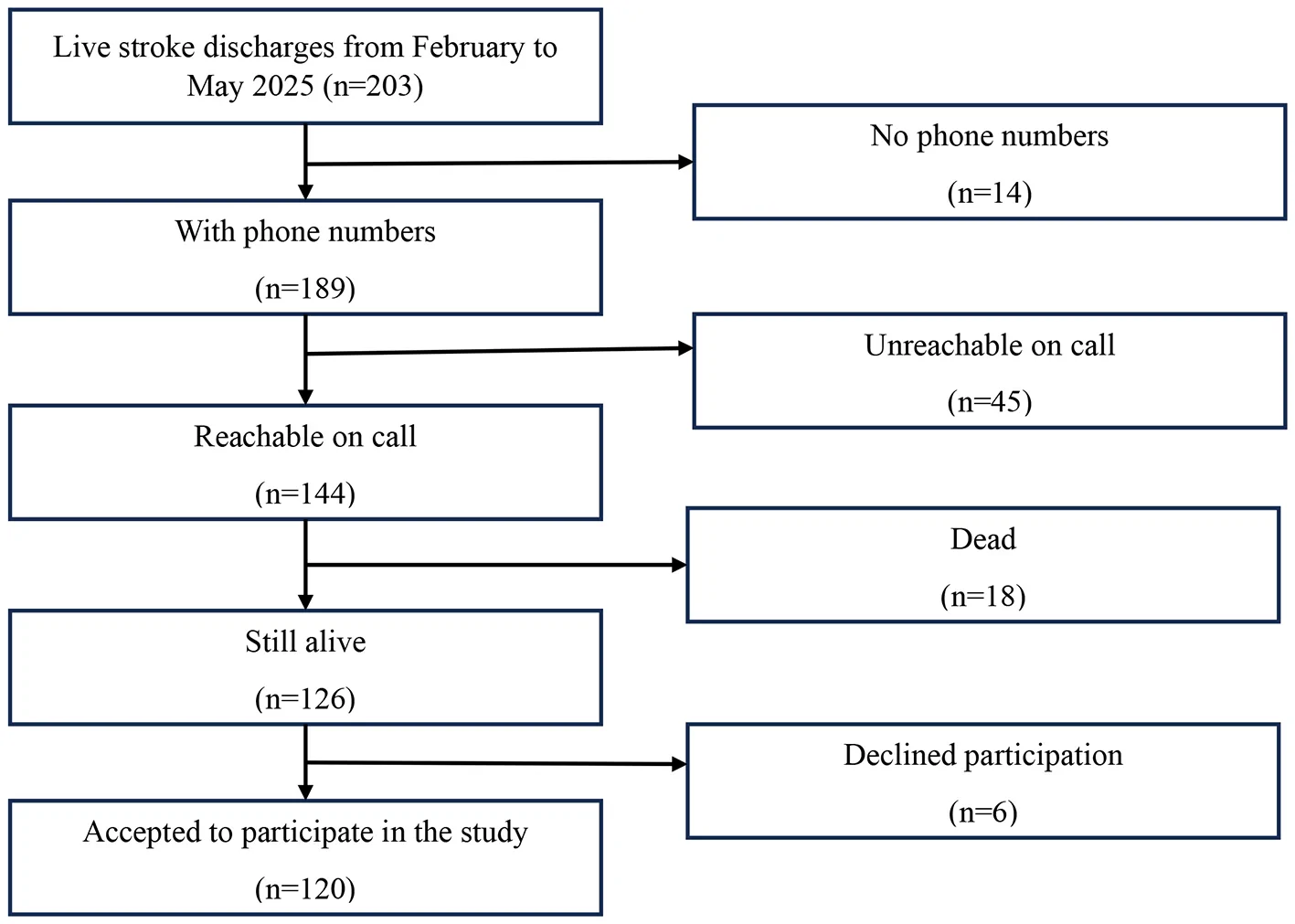
Participant flow for the study.

Of 203 stroke patients discharged alive from the Mulago National Referral Hospital Neurology unit between February and May 2025, only 126 patients were both alive and reachable by phone at the time of the study. Of these, 120 accepted to participate, yielding a response rate of 95.2% (120/126; Figure 1). Among the 120 participants, the mean age (SD) was 58.4 years (±14.6), 55% (66/120) of the participants were female, with 55.8% (67/120) married and 44.2% (53/120) had attained a primary level of education. Slightly over half (53.3%, 64/120) of the study participants resided in urban areas and 55.8% (67/120) had a high socio-economic status. Details of the socio-demographic characteristics are shown in Table 1.

**Table 1:** Univariate analysis.

Variables	Frequency (*n*)	Percentage (%)
**Overall**	120	100.0
Age (years)		
21–44	14	11.7
45–60	57	47.5
>60	49	40.8
Sex		
Female	66	55.0
Male	54	45.0
Highest education level attained		
None	27	22.5
Primary	53	44.17
Secondary	24	20.0
Tertiary	16	13.3
Marital status		
Divorced/separated	11	9.2
Married	67	55.8
Never married	13	10.8
Widowed	29	24.2
Region		
Central	94	78.3
East	12	10.0
North	4	3.3
West	10	8.3
Religion		
Born again	7	5.8
Anglican	22	18.3
Catholic	43	35.8
Muslim	23	19.2
Protestant	23	19.2
Seventh-day adventist	2	1.7
Type of residence		
Rural	64	53.3
Urban	56	46.7
Socio-economic status		
Low	53	44.2
High	67	55.8

The in-depth interviews involved six participants who were purposively selected from those who had attended the telephone interviews, with priority given to individuals who had attended stroke rehabilitation services multiple times and were willing to provide further information regarding their experiences of accessing stroke rehabilitation services. The IDI participants had a mean age of 47.3 (SD 16.8), the majority were male (66.7%, 4/6), married (83.3%, 5/6), and resided in urban areas (66.7%, 4/6).

### 3.2 Access to stroke rehabilitation services for patients discharged from the Mulago National Referral Neurology unit

“*Thirteen participants who self-reported no knowledge of the services and were unable to complete the multidimensional access tool were excluded from further analysis, although their demographic characteristics were retained in the descriptive analysis*.”

**Figure 2:**
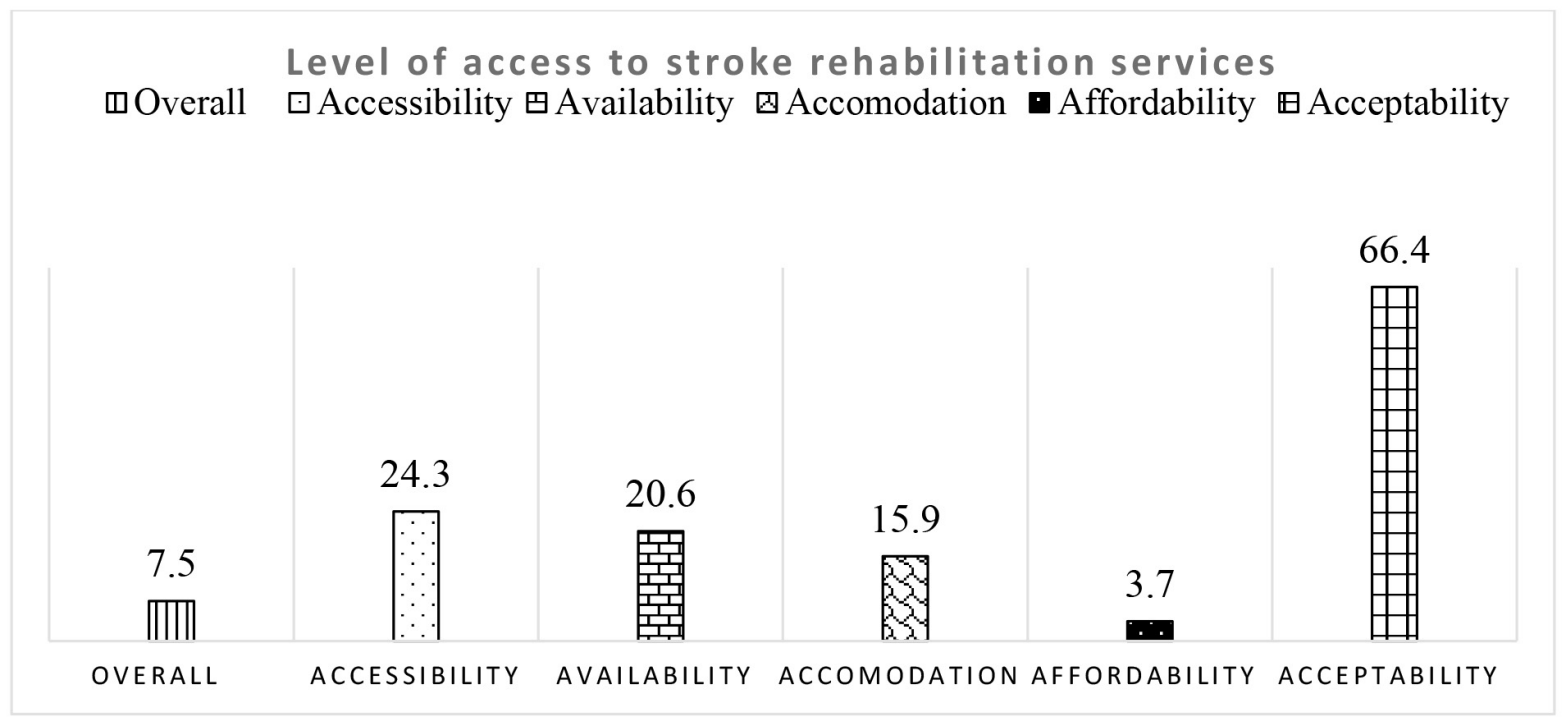
Bar chart illustrating the level of access across different dimensions.

Overall, only 7.5% (95% CI: 2.5%-12.5%) of participants (8/107) had high access to stroke rehabilitation services. However, this level varied across the different dimensions of access as shown in Figure 2.

Acceptability had the highest proportion of participants that scored 80% and above (66.4%, 71/107), while affordability was the lowest (3.7%, 4/107). In-depth interviews revealed negative experiences related to the cost of care and the patient's ability to afford this cost. The financial demands of rehabilitation were a recurring concern among participants, who reported paying for each physiotherapy session in addition to covering the cost of ongoing medications. For some, these combined expenses became unsustainable and led to the discontinuation of care.

“*It is very difficult for low-income people to afford the rehabilitation services, just that when it comes to saving a life, you have no choice, even if it costs a lot of money*” *(IDI 3, age 52 years, Male)*.

“*There was a guy [healthcare worker] we had found, but he was too expensive. He charged 80,000 UGX (approximately USD22) for every session, each lasting 2 h. He used to come for some days, but we eventually stopped using his services. We stopped most services [rehabilitation care] because of the expenses. Even the medication that he is on costs 85,000 UGX (approximately USD 24) weekly, and he is still on all the medication. So, if you add another 80,000 UGX for the physiotherapy, it becomes too overwhelming*” *(IDI 5, age 75 years, Male)*.

Furthermore, access to stroke rehabilitation services was mainly dependent on support from family and friends. Many patients noted that their relatives not only facilitated transport to health facilities but also helped cover treatment costs.

“*When I had now come out from the hospital, my friends and family would take me here and there for that kind of treatment [meaning rehabilitation]. Those who assisted me did pay, like my family and siblings' support, I would say, because it's a big family, along with friends*,” (*IDI 4, age 38 years, Male)*.

In the accessibility domain, 24.3% (26/107) of participants scored ≥80%, with a median score of 50% (IQR: 30–75). From in-depth interviews, patients reported a variety of transport challenges faced while accessing stroke rehabilitation services. Several stroke patients reported living far from health facilities that provide stroke rehabilitation services. The long travel distances made consistent clinic attendance difficult, leading some patients to discontinue therapy or resort to alternative approaches such as self-care at home.

“We *did just like two sessions of rehabilitation, and the distance became a problem for me*” *(IDI 5, age 75 years, Male)*.

Even participants who lived relatively close to rehabilitation centers also faced challenges related to transport. Many lacked appropriate means of transport, especially those with health vulnerabilities.

“*As you can see, the patient does not stay far from the hospital, but I cannot expose her to the wind [meaning a strong breeze], so we cannot use boda-bodas since, when it's too windy, she vomits*. *If you have transportation, there is no problem, but the issue is the means of transport and the money*” *(IDI 1, age 36 years, Female)*.

Some stroke patients reported incurring high transportation expenses when traveling to rehabilitation facilities. In some cases, the financial burden extended to family members, prompting patients themselves to contribute toward the costs.

“*My sister comes and drives me to the hospital, and then brings me back home. Of course, it's expensive. Sometimes she tells me she doesn't have fuel, and since I am the one in need, I have to contribute some money. So, she ends up sending the driver instead*” *(IDI 2, age 45 years, Male)*.

In the accommodation domain, 15.9% (17/107) of participants scored ≥80%, with a median score of 55% (IQR: 40–65). From the in-depth interviews, patients shared experiences regarding how stroke rehabilitation services were organized vis-à-vis their expectations and needs. Some participants reported that rehabilitation services at certain health facilities were only available during morning hours. Patients who arrived later in the day were not attended to, even if healthcare workers were still present at the facility.

“*When we returned to the hospital, they first inserted the tube, and afterwards we were told to slope down [to the rehabilitation department]. But by the time we got there, they had already finished. We went back another day and still found that they were done. We did not know that they [rehabilitation staff] are only available in the morning*” (*IDI 1, age 36 years, Female)*.

Participants also reported long waiting periods at hospitals before receiving rehabilitation services. Some described a lack of clear direction within the hospital system, resulting in delays and, in some instances, missed appointments.

“*We wait for a really long time. This time around, we were disturbed, and we left Mulago at around 6 p.m.……. We didn't get to see the doctor because time had passed. They kept sending us from one place to another. You go to one side, and when you return, they send you somewhere else. You don't even know where they are sending you, most of the time you need someone to direct you, because you don't know exactly where to find what you are being sent for, and often, there is no one to help you*” *(IDI 6, age 34 years, Female)*.

Patients accessing private rehabilitation services reported more organized and timely care, largely facilitated by appointment scheduling.

“*Usually, he would assign me a specific time to come. There was a time I went without informing him and found other people already there. He explained that I needed to come at the time he had given me because he also allocates appointments to other patients. Once in a while, he would even visit them at their homes, just like he did for me sometimes*” *(IDI 4, age 38 years, Male)*.

Due to accessibility barriers, some patients resorted to conducting rehabilitation exercises at home. These sessions were often guided by family members, friends with medical knowledge, or instructional videos found online.

“*It is us who try and help her do the exercises. We have a friend who is a doctor; she's the one who sometimes tells us, ‘Do this, try that.'*” *(IDI 6, age 34 years, Female)*.

In the availability domain, 20.6% of participants scored ≥80%. Participants reported limited availability of stroke rehabilitation services within their communities, with difficulty identifying nearby facilities or providers.

“*I have never seen any here [rehabilitation services], and I have never heard of them. I asked so much about it when we had just come back home [from the hospital]. It's been three months now; this is the fourth, and I still haven't seen anything. If I had gotten someone who could provide those services, I would have asked them to come and help us with those services, but I haven't got anyone*” *(IDI 1, age 36 years, Female)*.

However, some patients reported that in some hospitals, healthcare workers were consistently available and equipped with the necessary tools to support recovery.

“*They [healthcare workers] are always available, and before starting the exercises, they first review the medication you've been taking and assess your progress since the problem began. They even have machines to awaken the dead cells*” *(IDI 2, age 45 years, Male)*.

Supplementary Table 2 presents the distribution of access to stroke rehabilitation services across socio-demographic characteristics, based on the five dimensions of access (accessibility, availability, accommodation, affordability, and acceptability). High access was defined as a score ≥80% per dimension, and low access as < 80%. Higher access was reported among younger participants, urban residents, those of higher socio-economic status, and married individuals compared with their counterparts. Additionally, Table 2 shows a summary of themes and subthemes, categorized into negative and positive experiences faced while accessing stroke rehabilitation services.

**Table 2:** Subthemes and themes of experiences faced while accessing stroke rehabilitation services.

Category	Theme	Subtheme	Illustrative quotations
Negative experiences	Transport challenges	Long distance to the facility	*We did just like two sessions of rehabilitation, and the distance became a problem for me*
		Lack of transport and money	*As you can see, the patient does not stay far from the hospital, but I cannot expose her to the wind [meaning a strong breeze], so we cannot use boda-bodas since, when it's too windy, she vomits*. *If you have transportation, there is no problem, but the issue is the means of transport and the money*
		High transport costs	*My sister comes and drives me to the hospital, and then brings me back home. Of course, it's expensive. Sometimes she tells me she doesn't have fuel, and since I am the one in need, I have to contribute some money. So, she ends up sending the driver instead*
	High cost of care	High costs of physiotherapy and medication	*It is very difficult for low-income people to afford the rehabilitation services, just that when it comes to saving a life, you have no choice, even if it costs a lot of money*
		Dependence on family	*When I had now come out from the hospital, my friends and family would take me here and there for that kind of treatment [meaning rehabilitation]. Those who assisted me did pay, like my family and siblings' support, I would say, because it's a big family, along with friends*
	Organization of stroke rehabilitation	Limited working hours of healthcare workers	*When we returned to the hospital, they first inserted the tube, and afterwards we were told to slope down [to the rehabilitation department]. But by the time we got there, they had already finished. We went back another day and still found that they were done. We did not know that they [rehabilitation staff] are only available in the morning*
		Long waiting time and inefficient patient flow	*We wait for a really long time. This time around, we were disturbed, and we left Mulago at around 6 p.m.……. We didn't get to see the doctor because time had passed. They kept sending us from one place to another. You go to one side, and when you return, they send you somewhere else. You don't even know where they are sending you, most of the time you need someone to direct you, because you don't know exactly where to find what you are being sent for, and often, there is no one to help you*
		Shift to self-care	*It is us who try and help her do the exercises. We have a friend who is a doctor; she's the one who sometimes tells us, “Do this, try that”*
		Lack of physiotherapists in the community	*I have never seen any here [rehabilitation services], and I have never heard of them. I asked so much about it when we had just come back home [from the hospital]. It's been three months now; this is the fourth, and I still haven't seen anything. If I had gotten someone who could provide those services, I would have asked them to come and help us with those services, but I haven't got anyone*
Positive experiences	Organization of stroke rehabilitation services	Effective appointment system	*Usually, he would assign me a specific time to come. There was a time I went without informing him and found other people already there. He explained that I needed to come at the time he had given me because he also allocates appointments to other patients. Once in a while, he would even visit them at their homes, just like he did for me sometimes*
	Adequacy of stroke rehabilitation services	Availability of medical equipment and HCWs at the hospital	*They [healthcare workers] are always available, and before starting the exercises, they first review the medication you've been taking and assess your progress since the problem began. They even have machines to awaken the dead cells*

## 4 Discussion

This study aimed to assess access to stroke rehabilitation services and explore the experiences of patients discharged from the neurology unit of Mulago National Referral Hospital.

The small proportion of participants who achieved high access indicated that a significant proportion of stroke patients' face challenges in accessing necessary rehabilitation services after discharge from the MNRH-Neurology unit. These results are consistent with earlier studies conducted in Uganda, which have qualitatively reported similarly low levels of access to stroke rehabilitation services (Blixen et al., 2017; Nakibuuka et al., 2021; Timm et al., 2023).

Using Penchansky and Thomas's multidimensional model of access, affordability was the most affected dimension, indicating that post-discharge stroke rehabilitation costs exceeded patients' ability to pay. This contributed to the discontinuation of facility-based rehabilitation and a shift to self- and home-based care, as reported in the IDIs. Similar findings have been reported in Ghana, where financial constraints were a key barrier to accessing stroke rehabilitation services. In addition to therapy costs, patients also incurred expenses for diagnostic tests, medications, and assistive devices (Baatiema et al., 2021).

Following affordability was the accommodation of the stroke rehabilitation services. There was inadequate organization of stroke rehabilitation services vis-à-vis the patient's needs. The organization of the services only favors stroke patients who can travel to health facilities on time and arrive there in the morning. Long waiting times were also a concern. However, this can be explained by IDI findings, which included prolonged processes and multiple service points that require stroke patients to visit several departments to address different aspects of their condition. Furthermore, the limited number of trained rehabilitation personnel in the Uganda health care system could further explain the delays in service provision and limited operating hours.

In the dimension of availability, stroke patients reported significant challenges in locating facilities that offer rehabilitation services within their communities. Most stroke rehabilitation care in the Uganda healthcare system is provided at the national and regional referral hospitals (MOH, 2022), with no further extension to communities. This finding is consistent with Nakibuuka et al. (2021), who reported a lack of community stroke rehabilitation centers in Uganda.

Regarding accessibility, stroke patients encountered difficulties in physically reaching rehabilitation facilities. In Uganda, majority of the population resides in the rural areas (UBOS, 2024) yet, most health facilities that can provide stroke rehabilitation are located in urban centers. The poor road infrastructure in most parts of the country, and limited transport means, make it more difficult for patients to access these services. These findings align with previous studies, which also identified transportation barriers and the financial burden of travel as major concerns affecting access to stroke rehabilitation (Koh et al., 2014; Mlenzana et al., 2018).

The relatively high score in acceptability of stroke rehabilitation services may be explained by the limited autonomy stroke patients have in choosing their rehabilitation providers. Given their strong need for rehabilitation services, patients are often compelled to accept care regardless of the provider's characteristics. As a result, the acceptability score may reflect the patient's willingness to receive care from any available provider.

Findings from this study are consistent with the rehabilitation call 2030 (Gimigliano and Negrini, 2017), which acknowledges the limited and failed capacity of various countries to provide rehabilitation that adequately addresses the needs of the population. This is explained by factors like under-prioritization by governments, absence of rehabilitation policies, inadequate funding, limited evidence for met and unmet rehabilitation needs, insufficient number and skills of rehabilitation professionals, absence of rehabilitation facilities and equipment, and lack of integration into health systems.

In Uganda, this underscores the need for political commitment and policy reforms to strengthen financial risk protection for essential stroke rehabilitation services and assistive devices, and to invest in the training, recruitment, and equitable distribution of rehabilitation professionals beyond national referral hospitals to regional, district, and community levels. Policy frameworks should support task-sharing models, enabling trained mid-level providers and community health workers to deliver basic rehabilitation interventions under supervision. In addition, stronger referral, discharge planning, and follow-up systems are required to ensure continuity of rehabilitation care after hospital discharge, as recommended by WHO.

Potential interventions that can be adapted in this context to improve access to post discharge stroke rehabilitation services could include home-based and community led rehabilitation models, which have been found effective (Ru et al., 2016) even in resource limited settings (Ali et al., 2025; Basheikh and Badahdah, 2025). Under these models, trained caregivers and community health workers could deliver standardized rehabilitation exercises with periodic supervision from rehabilitation professionals. These models would reduce the need for stroke patients to travel frequently to health facilities, reducing transport challenges, while improving the availability and accommodation of stroke rehabilitation services.

Furthermore, tele-rehabilitation and mobile health (mHealth) interventions offer opportunities to extend the reach of rehabilitation services, especially to rural populations (Prvu Bettger et al., 2019). Public-private partnerships could also be utilized and contracted to expand affordable rehabilitation services, especially in underserved areas.

## 5 Study strengths and limitations

### 5.1 Strengths

The study employed a mixed-methods approach, integrating both quantitative and qualitative research methods. The qualitative component helped explain the quantitative findings, hence enriching the interpretation of results.

Additionally, the study employed a multidimensional approach to measure access to stroke rehabilitation services. This enabled the identification and comparison of specific dimensions influencing access, thereby highlighting which aspects had the greatest impact on patients' ability to access care.

### 5.2 Limitations

Consecutive sampling may limit the generalizability of these findings; however, the study was conducted at a national referral hospital serving patients from across Uganda, suggesting some sample diversity. The exclusion of deceased and unreachable participants may have introduced selection bias, although its magnitude could not be assessed due to limited information on non-respondents. The study is also subject to recall and survival bias, and self-reported access to rehabilitation services could not be independently verified. Lastly, the small sample size could also limit the generalizability of these study findings. Future studies should use multi-site designs and larger, more representative samples to improve generalizability and enable robust estimation of factors associated with access to post-discharge stroke rehabilitation services.

## 6 Conclusions

Access to stroke rehabilitation services following discharge from the Neurology Unit of Mulago National Referral Hospital was very low (7.5%) and was primarily constrained by high costs of care, transport barriers, and limitations in service availability and organization. Affordability was the most affected dimension compared with other access domains.

## 7 Recommendations

To improve access to post-discharge stroke rehabilitation services, the following are recommended:

Subsidize rehabilitation services: given that affordability was the most constrained dimension, the Uganda Ministry of Health (MoH) and implementing partners should introduce subsidized community-based stroke rehabilitation services to reduce out-of-pocket costs.

Extend service hours: rehabilitation units should extend operational hours to accommodate patients who are unable to attend morning sessions, thereby improving service accommodation.

Strengthen caregiver training: the MoH and implementing partners should develop structured pre-discharge and follow-up training to equip caregivers with basic rehabilitation skills, supporting more affordable home-based care.

Decentralize rehabilitation services: community-based rehabilitation points should be established through collaboration between the MoH, local governments, implementing partners, and rehabilitation departments to improve service availability and accessibility, supported by capacity building and provision of essential equipment.

Conduct further research: future studies should evaluate the effectiveness and long-term outcomes of home-based rehabilitation on functional recovery among stroke survivors.

## Data Availability

The original contributions presented in the study are included in the article/Supplementary material, further inquiries can be directed to the corresponding author/s.
