# Supplementary material for: Access to stroke rehabilitation services for patients discharged from the Mulago National Referral Hospital Neurology unit, Kampala—Uganda: a mixed-methods study
Source: Front Stroke. 2026 Aug 24;5:1913292. doi: 10.3389/fstro.2026.1913292 (PMC13547042; doi:10.3389/fstro.2026.1913292)
Supplement: Supplementary file 1 [file Table_1.pdf]

1. **Supplementary Table 1:** This table shows the independent variables of the study, their type, and measurement.

Supplementary Table 1: Type and measurement of independent variables

**Variable**	**Variable type**	**Measurement**
Age	Continuous variable	What is your current age in completed years as of your last birthday?
Sex	Categorical	What is your sex?<br>0=Male 1=Female
Ethnicity	Categorical	What is your ethnicity?<br>Baganda, Banyankole, Basoga, Bakiga, Others (please specify)
Marital status	Categorical	What is your marital status?<br>0=Married 1=Single 2=Divorced 3=Separated 4=Widowed
Level of education	Categorical	What is your highest level of education attained?<br>0=Primary 1= secondary 3=tertiary
Area of residence	Categorical	What is your current area of residence? (*district, sub-county, parish*)
Type of residence	Categorical	Was determined based on official designations<br>0=Urban 1=Rural
Religion	Categorical	What is your religious affiliation? 0=Anglican 1=Muslim 2=protestant

		3=Catholic 4=Seventh-Day Adventist 5=Orthodox Others (please specify) …
Household wealth	Categorical	A wealth index was used to classify individuals into quintiles of SES<br>0=Lowest 1=First 2=Second 3=Third 4=Fourth 5=Fifth
Attendance of stroke rehabilitation services	Categorical	Following discharge from the MNRH-Neurology unit, have you attended any rehabilitation sessions for stroke? (Yes/No)<br>0=No 1=Yes
Distance from home to health facility	Categorical	How would you rate the distance between your area of residence and the nearest health facility providing stroke rehabilitation services?<br>*(1=very far 2=far 3=neutral 4=near 5=very near)*
Time to reach the rehabilitation health facility	Ordinal	How satisfied are you with the time it takes to reach this facility?<br>*(1=very dissatisfied 2=dissatisfied) 3=neutral 4=satisfied 5=very satisfied)*
Transport costs to rehabilitation facility	Ordinal	How satisfied are you with the cost of traveling to this facility?<br>*(1=very dissatisfied 2=dissatisfied) 3=neutral 4=satisfied 5=very satisfied)*
Appropriate means of transport	Ordinal	How satisfactory is the means of transport you use to travel for stroke rehabilitation services?

		(1=very dissatisfied 2=dissatisfied) 3=neutral 4=satisfied 5=very satisfied)
Appointment system	Ordinal	How satisfied are you with the time spent at the facility before receiving rehabilitation service? *(1=very dissatisfied 2=dissatisfied) 3=neutral 4=satisfied 5=very satisfied)*
Telephone system	Ordinal	How easy is it to receive rehabilitation services through the telephone system? *(1=very difficult 2=difficult 3=neutral 4=easy 5=very easy)*
Community rehabilitation care	Ordinal	How easy is it to receive rehabilitation care from home? *(1=very difficult 2=difficult 3=neutral 4=easy 5=very easy)*
Cost of rehabilitation care	Ordinal	How satisfied are you with the cost of receiving stroke rehabilitation services? *(1=very dissatisfied 2=dissatisfied) 3=neutral 4=satisfied 5=very satisfied)*
Health insurance coverage for rehabilitation care	Ordinal	How satisfactory is the insurance coverage in covering rehabilitation costs? *(1=very dissatisfied 2=dissatisfied) 3=neutral 4=satisfied 5=very satisfied)*
Family financial support	Ordinal	How satisfied are you with the family and social support you receive while meeting rehabilitation bills?

		(1=very dissatisfied 2=dissatisfied 3=neutral 4=satisfied 5=very satisfied)
Availability of a health facility providing stroke rehabilitation services	Ordinal	How easy is it to locate a facility providing stroke rehabilitation services within your area of residence?<br>*(1=very difficult 2=difficult 3=neutral 4=easy 5=very easy)*
Number of rehabilitation health workers	Ordinal	How easy is it to meet a health worker specialized in providing stroke rehabilitation services?<br>*(1=very difficult 2=difficult 3=neutral 4=easy 5=very easy)*
Availability of stroke therapeutic equipment	Ordinal	How satisfied are you with the availability of therapeutic equipment and supplementary treatments at the facility?<br>*(1=very dissatisfied 2=dissatisfied) 3=neutral 4=satisfied 5=very satisfied)*
The attitude of stroke patients towards the individual characteristics of HCWs providing rehabilitation	Ordinal	How would you rate your attitude towards the individual characteristics of health workers providing stroke rehabilitation services?<br>***(**1=very bad 2=bad 3=neutral 4=good 5=very good**)***

The attitude of stroke patients towards the individual characteristics of HCWs providing rehabilitation	Ordinal	How satisfied are you with the experience of health workers in providing stroke rehabilitation services?<br>*(1=very dissatisfied 2=dissatisfied 3=neutral 4=satisfied 5=very satisfied)*
