# Supplementary material for: Access to stroke rehabilitation services for patients discharged from the Mulago National Referral Hospital Neurology unit, Kampala—Uganda: a mixed-methods study
Source: Front Stroke. 2026 Aug 24;5:1913292. doi: 10.3389/fstro.2026.1913292 (PMC13547042; doi:10.3389/fstro.2026.1913292)
Supplement: Supplementary file 2 [file Table_2.pdf]

2. **Supplementary Table 2** shows the Distribution of the level of access to stroke rehabilitation services according to socio-demographic factors

| Variable | Overall | | Accessibility | | Availability | | Accommodation | | Affordability | | Acceptability | |
|---|---|---|---|---|---|---|---|---|---|---|---|---|
| Overall | **Low (n=99)** | **High (n=8)** | **Low** | **High** | **Low** | **High** | **Low** | **High** | **Low** | **High** | **Low** | **High** |
| Age | | | | | | | | | | | | |
| <45 | 11 (84.6) | 2 (15.4) | 9(69.2) | 4(30.8) | 7(53.8) | 6(46.2) | 10(76.9) | 3 (23.1) | 12 (92.3) | 1 (7.7) | 3(23.1) | 10(76.9) |
| 45-60 | 48 (96.0) | 2 (4.0) | 35(70.0) | 15(30.0) | 41(82.0) | 9(18.0) | 42(84.0) | 8(16.0) | 48(96.0) | 2(4.0) | 14(28.0) | 36(72.0) |
| >60 | 40 (90.9) | 4 (9.1) | 37(84.1) | 7(15.9) | 37(84.1) | 7(15.9) | 38(86.4) | 6(13.6) | 43(97.7) | 1(2.3) | 19(43.2) | 25(56.8) |
| Sex | | | | | | | | | | | | |
| Female | 60 (92.3) | 5 (7.7) | 48(73.8) | 17(26.2) | 51(78.5) | 14(21.5) | 56(86.2) | 9(13.8) | 63(96.9) | 2(3.1) | 24(36.9) | 41(63.1) |
| Male | 39 (92.9) | 3 (7.1) | 33(78.6) | 9(21.4) | 34(80.9) | 8(19.1) | 34(80.9) | 8(19.1) | 40(95.2) | 2(4.8) | 12(28.6) | 30(71.4) |
| Education | | | | | | | | | | | | |
| None | 24 (92.3) | 2 (7.7) | 23(88.5) | 3(11.5) | 22(84.6) | 4(15.4) | 22(84.6) | 4(15.4) | 24(92.3) | 2(7.7) | 16(61.5) | 10(38.5) |
| Primary | 43 (91.5) | 4 (8.5) | 29(61.7) | 18(38.3) | 37(78.7) | 10(21.3) | 41(87.2) | 6(12.8) | 45(95.7) | 2(4.3) | 9(19.2) | 38(80.8) |
| Secondary | 20 (95.2) | 1 (4.8) | 17(81.0) | 4(19.0) | 17(80.9) | 4(19.1) | 18(85.7) | 3(14.3) | 21(100.0) | 0(0.0) | 6(28.6) | 15(71.4) |
| Tertiary | 12 (92.3) | 1 (7.7) | 12(92.3) | 1(7.7) | 9(69.2) | 4(30.8) | 9(69.2) | 4(30.8) | 13(100.0) | 0(0.0) | 5(38.5) | 8(61.5) |
| Marital status | | | | | | | | | | | | |
| Married | 52 (91.2) | 5 (8.8) | 46(80.7) | 11(19.3) | 43(75.4) | 14(24.6) | 46(80.7) | 11(19.3) | 54(94.7) | 3(5.3) | 17(29.8) | 40(70.2) |
| Not married | 47 (94.0) | 3 (6.0) | 35(70.0) | 15(30.0) | 42(84.0) | 8(16.0) | 44(88.0) | 6(12.0) | 49(98.0) | 1(2.0) | 19(38.0) | 31(62.0) |
| Religion | | | | | | | | | | | | |
| Catholic | 32 (88.9) | 4 (11.1) | 28(77.8) | 8(22.2) | 30(83.3) | 6(16.7) | 31(86.1) | 5(13.9) | 34(94.4) | 2(5.6) | 12(33.3) | 24(66.7) |
| Anglican | 17 (85.0) | 3 (15.0) | 17(85.0) | 3(15.0) | 12(60.0) | 8(40.0) | 12(60.0) | 8(40.0) | 18(90.0) | 2(10.0) | 8(40.0) | 12(60.0) |
| Others | 50 (98.0) | 1 (2.0) | 36(70.6) | 15(29.4) | 43(84.3) | 8(15.7) | 47(92.2) | 4(7.8) | 51(100.0) | 00.0) | 16(31.4) | 35(68.6) |
| Type of residence | | | | | | | | | | | | |
| Rural | 55 (96.5) | 2 (3.5) | 47(82.5) | 10(17.5) | 52(91.2) | 5(8.8) | 52(91.2) | 5(8.8) | 56(98.3) | 1(1.7) | 20(35.1) | 37(64.9) |
| Urban | 44 (88.0) | 6 (12.0) | 34(68.0) | 16(32.0) | 33(66.0) | 17(34.0) | 38(76.0) | 12(24.0) | 47(94.0) | 3(6.0) | 16(32.0) | 34(68.0) |
| Socio-economic status | | | | | | | | | | | | |
| Low | 46 (93.9) | 3 (6.1) | 41(83.7) | 8(16.3) | 40(81.6) | 9(18.4) | 44(89.8) | 5(10.2) | 48(98.0) | 1(2.0) | 21(42.9) | 28(57.1) |
| High | 53 (91.4) | 5 (8.6) | 40(69.0) | 18(31.0) | 45(77.6) | 13(22.4) | 46(79.3) | 12(20.7) | 55(94.8) | 3(5.2) | 15(25.9) | 43(74.1) |
